# Advances in the treatment of acne scars

**DOI:** 10.3389/fmed.2025.1643035

**Published:** 2025-08-29

**Authors:** Meng Zhang, Chunmei Liu, Shengni Zhang, Ruiqi Chu, Xiangxiang Ren

**Affiliations:** ^1^Department of Dermatology, Affiliated Hospital of Hebei University, Baoding, Hebei, China; ^2^Affiliated Hospital of Hebei University, Baoding, Hebei, China; ^3^Department of General Surgery, Affiliated Hospital of Hebei University, Baoding, Hebei, China

**Keywords:** acne scars, atrophic acne scars, combined modality therapy, hypertrophic scars, laser therapy

## Abstract

Acne vulgaris and its resulting scars represent common clinical dermatological concerns. Even after the resolution of active lesions, scars can cause significant psychological distress and severely impair patients' quality of life. In recent years, advancements in medical technology have led to the clinical application of various innovative therapies for acne scar management, with the primary goals of effectively improving both the aesthetic appearance and functionality of the scars. Treatment strategies continue to be refined—ranging from traditional physical modalities (such as microneedling) to modern energy-based devices (such as fractional lasers)—to balance efficacy and safety. Current research focuses on developing combination therapy approaches. The integration of chemical peels, laser technologies, and dermal fillers is emerging as a key trend for the future, aiming to enhance treatment outcomes while reducing treatment burden.

## 1 Introduction

Acne vulgaris is a highly prevalent chronic inflammatory dermatosis. Epidemiological studies indicate that ~85% of adolescents are affected by this condition (1). Its pathogenesis involves both inflammatory and non-inflammatory lesions, characterized by an imbalance in the expression of matrix metalloproteinases (MMPs) and tissue inhibitors of metalloproteinases (TIMPs), as well as dysregulation of the transforming growth factor-beta 1 (TGF-β1) signaling pathway. These mechanisms contribute to the breakdown of dermal structure and aberrant repair processes, ultimately leading to scar formation (1). Acne scars predominantly localize to facial regions and can induce long-term psychological sequelae including self-esteem impairment, social avoidance, and reduced quality of life. This article systematically reviews interventional strategies—such as chemical peels, laser therapy, dermal filler injections, and surgical procedures—to provide evidence-based guidance for clinical practice.

### 1.1 Classification and epidemiology

**Figure 1:**
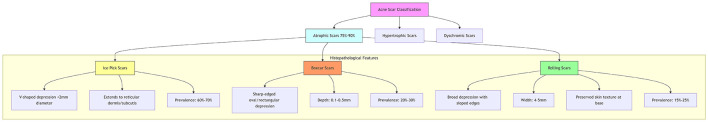
Morphological classification of atrophic acne scars.

Based on morphological characteristics, acne scars are classified into three categories: atrophic, hypertrophic, and dyschromic (2). Among these, atrophic scars account for 75%−90% of cases, with an incidence approximately three times higher than hypertrophic scars (2). According to anatomical features, atrophic scars are further subdivided into: ice pick scars: V-shaped depressions < 2 mm in diameter extending to the reticular dermis or subcutaneous tissue, typically refractory to superficial epidermal treatments (2). Boxcar scars: sharply demarcated oval or rectangular depressions with depths of 0.1–0.5 mm. Rolling scars: broad depressions (4–5 mm wide) with sloped edges and preserved skin texture at the base, creating an undulating appearance. Epidemiological analyses indicate the following prevalence among atrophic scars: ice pick (60%−70%), boxcar (20%−30%), and rolling scars (15%−25%) (2). Given this heterogeneity in acne scarring characteristics, distinct therapeutic strategies are required for different subtypes. As illustrated in Figure 1, the three primary subtypes of atrophic scars exhibit distinct morphological characteristics and epidemiological distributions.

This study integrates evidence-based medical data to propose a stratified treatment algorithm tailored to scar morphology, aiming to optimize clinical outcomes.

## 2 Chemical peeling

Chemical peeling induces controlled removal of pathological epidermal and partial dermal tissues, promoting epidermal regeneration for scar repair. Epidermal regeneration typically initiates within 24 h post-procedure and completes within 7–10 days. Neocollagenesis achieves skin tightening and surface smoothing through collagen and glycosaminoglycan deposition (3). Chemical peel procedures carry inherent risks, including post-inflammatory hyperpigmentation and scar neogenesis, with the incidence of these complications correlating positively with the concentration of the peeling agent. For superficial scars, epidermal-level intervention is achievable using agents such as glycolic acid, lactic acid, salicylic acid, or Jessner's solution (a compounded formulation of resorcinol, salicylic acid, and lactic acid in ethanol), which concurrently address associated post-inflammatory hyperpigmentation. Medium-depth scars, requiring penetration to the papillary dermis, are effectively managed with trichloroacetic acid (TCA) at concentrations of 30%−40%. Deep atrophic scars necessitate aggressive intervention targeting the reticular dermis, typically employing 50% TCA or phenol; however, these higher-concentration agents carry significantly elevated risks of procedural complications (4).

### 2.1 Chemical reconstruction of skin scars (CROSS)

Focal application of 65%−100% TCA (TCA CROSS) selectively destroys scar tissue while stimulating collagen remodeling. Seventy percent TCA CROSS demonstrates significant improvement across all atrophic scar subtypes (including severe cases) (5), while the 100% concentration protocol serves as a cost-effective alternative for ice pick scars in dark-skinned patients—though remaining inferior to CO_2_ laser therapy in efficacy (6). Consequently, combination therapy integrating CROSS with lasers or microneedling is widely adopted to enhance outcomes while minimizing complications.

Although phenol has been largely phased out due to potential cardiotoxicity, modified-concentration CROSS techniques circumvent the need for cardiac monitoring. Studies indicate that 88% phenol CROSS combined with subcision and microneedling achieves consistent scar improvement (7). Optimizing peelant concentration may enhance efficacy while reducing adverse effects, suggesting phenol CROSS as a viable alternative to TCA CROSS in selected cases.

## 3 Energy-based devices

### 3.1 Laser therapy

Laser therapy utilizes monochromatic light to selectively target scar tissue, stimulating dermal fibroblast proliferation and promoting regeneration of collagen and elastic fibers, thereby repairing acne scars (8). This modality improves scar pigmentation, erythema, and textural depressions, clinically categorized as ablative and non-ablative systems.

#### 3.1.1 Ablative lasers

The 10,600-nm carbon dioxide (CO_2_) and 2,940-nm erbium-doped yttrium aluminum garnet (Er:YAG) lasers represent first-line treatments for atrophic acne scars, achieving overall efficacy rates up to 90%. Their mechanism involves selective photothermolysis of water molecules to vaporize scar tissue and induce collagen remodeling. Histological studies demonstrate significantly improved dermal collagen density, elastic fiber length, and skin texture with concomitant scar volume reduction following fractional Er:YAG and CO_2_ laser treatments (9). Gene expression analyses reveal that fractional CO_2_ lasers upregulate tissue remodeling genes [e.g., matrix metalloproteinase-3 (MMP-3)], enhancing fibroblast contraction and wound repair—peaking at day 1 post-treatment and subsiding by day 5 (10). Common adverse effects include persistent erythema, edema, and post-inflammatory hyperpigmentation (11). Pulse mode selection should be scar type-specific: long-pulse settings optimize efficacy for depressed scars, dual-mode Er:YAG lasers provide superior stability, while short-pulse protocols show limited efficacy for deep boxcar scars (12).

Multimodal procedural therapy (MMP) strategically balances therapeutic efficacy with patient safety through a sequential three-step approach. Treatment commences with high-energy fractional CO_2_ laser ablation, precisely targeting focal atrophic scars. This is followed by meticulous manual sculpting to refine the elevated borders of any hypertrophic scarring. The procedure concludes with low-energy fractional laser resurfacing applied to the surrounding peripheral tissue to promote seamless blending and rejuvenation. Clinical evidence suggests this integrated methodology significantly improves the appearance of facial atrophic scarring while concurrently reducing complication rates (13).

#### 3.1.2 Non-ablative lasers

The 1,540- and 1,550-nm erbium-doped fiber lasers stimulate neocollagenesis through selective photothermolysis while preserving the epidermis, making them suitable for mild-to-moderate atrophic scars (14). Long-term follow-up indicates superior efficacy and patient satisfaction with ablative Er:YAG lasers compared to non-ablative systems (15). Although less effective, non-ablative lasers offer shorter recovery times and fewer side effects. Concomitant application of mucopolysaccharide polysulfate cream may further enhance scar rehabilitation (16).

### 3.2 Picosecond lasers

Picosecond-domain pulse lasers demonstrate significant efficacy in treating pigmentary disorders (e.g., tattoos, ephelides), skin rejuvenation, and scar revision (17). Their ultrashort pulses generate laser-induced optical breakdown (LIOB), creating plasma vacuoles within the epidermis to selectively destroy target tissues. Studies confirm comparable efficacy between picosecond lasers and fractional CO_2_ lasers for acne scar improvement, with reduced post-procedural hyperpigmentation risk (18). Clinical data indicate significant scar volume reduction in 75% of patients after picosecond laser treatment, with no severe adverse events reported (19).

The 1,064-nm picosecond laser specifically addresses atrophic scars in Fitzpatrick skin types III-V, achieving 32%–45% scar volume reduction within 3 months (20).

When combined with microlens array (MLA) technology, the 1,064-nm picosecond laser generates high-density microthermal injury zones at the dermal-epidermal junction. This precision-targeted approach stimulates collagen remodeling without collateral tissue damage, concurrently improving scar depression, enlarged pores, and skin texture while shortening recovery time to 3–5 days (21).

### 3.3 Radiofrequency therapy

Radiofrequency (RF) therapy utilizes electrical current to generate controlled thermal effects within the dermis, stimulating collagen contraction and subsequent regeneration. This modality demonstrates a favorable safety profile, exhibiting lower adverse event rates compared to conventional laser therapies (22). Clinical RF systems are classified into three primary categories: monopolar RF, which demonstrates particular efficacy in treating active cystic scars, achieving documented clearance rates of 78%−85% in East Asian populations, specifically Japanese and Korean cohorts (22); bipolar RF; and fractional RF. The fractional approach promotes scar repair through the creation of minimally invasive, controlled thermal micro-injuries, a mechanism associated with lower complication rates than traditional laser systems.

Current evidence supports the feasibility of RF for acne scar treatment. However, further large-scale controlled studies are required to establish efficacy differentials vs. ablative/non-ablative lasers and identify optimal indications.

### 3.4 Fractional radiofrequency microneedling (FRMN)

Fractional radiofrequency microneedling (FRMN) combines mechanical injury with targeted thermal energy. Insulated needles (0.5–3.5 mm depth) deliver RF current directly to the dermis while preserving the epidermis, significantly reducing dyspigmentation risks in Fitzpatrick IV–VI skin. Three key mechanisms drive scar remodeling: (1) microchannel-induced neocollagenesis via platelet activation. (2) controlled thermal denaturation of fibrotic bands. (3) upregulation of HSP70/MMP-3 for extracellular matrix reorganization (23–25).

## 4 Dermal filler augmentation

### 4.1 Soft tissue fillers

Dermal fillers augment soft tissue volume, demonstrating optimal efficacy for pliable rolling or boxcar scars. Injectable agents include hyaluronic acid (HA), calcium hydroxylapatite (CaHA), and poly-L-lactic acid (PLLA; Table 1). These dermal filler agents may be employed either as standalone interventions or synergistically combined with subcision techniques to optimize the aesthetic improvement of atrophic acne scars. Hyaluronic acid (HA) enhances collagen formation within dermal fibroblasts, thereby improving scar appearance (26); however, treatment typically necessitates multiple sessions, and the longevity of results is variable. Calcium hydroxylapatite (CaHA) achieves sustained improvement, particularly in superficial atrophic variants such as rolling scars, often after a single injection, with clinical effects persisting for up to 12 months (27). Poly-L-lactic acid (PLLA) stimulates neocollagenesis, demonstrating notable efficacy for rolling scars and yielding >75% patient satisfaction at 24-month follow-up (28). Polymethylmethacrylate (PMMA), distinct from biodegradable options as a permanent filler, exhibits significant effectiveness in reducing the appearance of atrophic acne scars (29).

**Table 1:** Comparative analysis of dermal fillers for atrophic acne scar treatment: duration, indications, collagen stimulation, and skin type considerations.

**Filler**	**Duration**	**Best indication**	**Collagen stimulation**	**Skin tone restriction**
HA	6–12 months	Mild rolling scars	+	None
CaHA	12–18 months	Superficial rolling scars	++	None
PLLA	24+ months	Moderate-to-severe rolling scars	+++	None
PdLLA	18–24 months	Superficial boxcar/rolling scars	+++	None
PCL	24+ months	Deep boxcar/ice-pick scars	++++	Fitzpatrick III-VI
PMMA	Permanent	Deep atrophic scars	+	Use caution in dark skin

Hyaluronic acid (HA) enhances collagen formation within dermal fibroblasts, thereby improving scar appearance. Its efficacy varies significantly based on cross-linking status and injection techniques: non-cross-linked HA (low viscosity): primarily used in needle-free electro-pneumatic injection (EPI-HA). The high-velocity airflow drives HA particles into dermal microchannels, creating a “nanobullet cutting effect” that releases fibrotic tethers while volumizing scars. This approach shows optimal outcomes for severe rolling scars with minimal downtime (3–5 days) (30–34).

Cross-linked HA (high viscosity): provides structural support for deep volume loss. Studies confirm its collagen-stimulating effect via CD44 receptor activation, with effects lasting 6–12 months. A split-face RCT demonstrated >50% improvement in boxcar scars after a single injection (35).

Combination strategy: subcision + cross-linked HA yields superior results for fibrotic rolling scars compared to monotherapy (*p* < 0.01) (36).

Poly-D,L-lactic acid (PdLLA): PdLLA is a biphasic copolymer that combines immediate volumizing effects with long-term collagen stimulation. Unlike PLLA which requires reconstitution, PdLLA comes pre-dissolved for homogeneous dispersion. Clinical studies demonstrate 68%−75% improvement in rolling scars after two sessions, with effects persisting 18–24 months. Its unique rheology allows for precise intradermal placement in shallow boxcar scars (37).

Polycaprolactone (PCL): PCL microspheres (25–50 μm diameter) stimulate sustained neocollagenesis through controlled foreign-body reaction. A pivotal trial showed >70% scar improvement in Fitzpatrick IV–VI skin with minimal granuloma risk (>0.5%). Optimal for deep atrophic scars due to high elasticity modulus (G' >250 Pa), providing structural support against skin tension (38).

### 4.2 Autologous fat transplantation

Autologous fat grafting addresses severe atrophic scars through lipoaspiration from donor sites followed by fat injection into scar depressions. The procedure is typically preceded by subcision before fat transfer. However, this technique demands significant operator expertise and demonstrates variable graft retention rates, with optimal outcomes observed at 3 months postoperatively. Fat transplantation shows favorable efficacy for atrophic acne scars. Emerging evidence indicates that nanofat grafting achieves superior clinical improvement in acne scar treatment with a lower complication profile (39).

### 4.3 Platelet-rich products

#### 4.3.1 Platelet-rich plasma (PRP)

PRP injection utilizes autologous blood to treat acne scars. Containing high concentrations of growth factors, PRP stimulates regeneration of collagen and elastin. It demonstrates better efficacy for boxcar and rolling scars but has limited effectiveness against ice pick scars. Most studies indicate synergistic effects when PRP is combined with other modalities. PRP injections may reduce treatment intervals and accelerate acne scar rehabilitation.

Combination therapy with fractional CO_2_ lasers and PRP significantly improves scar revision outcomes while enhancing psychological wellbeing and quality of life in acne scar patients (40).

#### 4.3.2 Microneedling with PRP

When combined for atrophic acne scars, microneedling not only induces neocollagenesis but also enhances PRP absorption. PRP application upregulates protein synthesis, promoting collagen remodeling and accelerating wound healing.

#### 4.3.3 Platelet-rich fibrin (PRF)

As an advanced biomaterial, injectable PRF offers several advantages: simplified preparation and sustained release of growth factors that facilitate collagen remodeling and scar appearance improvement. Platelet-rich fibrin (PRF) establishes a resilient fibrin scaffold that facilitates the sustained release of growth factors over 7–14 days, contrasting sharply with the rapid cytokine depletion characteristic of platelet-rich plasma (PRP) (41). Clinically, the injection of PRF combined with microneedling demonstrates marked improvement in the appearance of atrophic acne scars, with the most pronounced outcomes observed for rolling scars, followed by boxcar scars (41). Therapeutically, PRF exhibits significant efficacy in acne scar management, coupled with a favorable profile characterized by minimal adverse effects and a relatively straightforward procedural technique.

### 4.4 Autologous bone marrow stem cells

Stem cells (SCs) possess the capacity to generate one or more specific tissue types. The clinical application of adult stem cells—specifically mesenchymal stem cells (MSCs)—in skin and scar repair has become clinically feasible. Bone marrow-derived stem cells can differentiate into various skin cell lineages, including keratinocytes and fibroblasts, thereby facilitating skin regeneration and scar improvement. Intradermal injection of autologous bone marrow stem cells demonstrates safety and efficacy across all subtypes of atrophic scars (42).

### 4.5 Autologous fibroblasts

Autologous fibroblast therapy is a novel, natural approach to repair dermal defects. Autologous fibroblasts are injected into atrophic acne scars; this procedure does not require allergy testing. These implanted fibroblasts produce collagen *in vivo*, thereby filling depressed scars and improving the clinical appearance of acne scarring. Intradermal injection of autologous fibroblasts represents a well-tolerated treatment modality for atrophic acne scars (43).

### 4.6 Botulinum toxin treatment

Botulinum toxin (BoNT) improves skin texture by inhibiting muscle fiber contraction at the scar edges, thereby reducing local tension. It concurrently modulates the TGF-β signaling pathway to promote orderly collagen deposition and suppresses the release of inflammatory factors (e.g., IL-6, TNF-α) (44). Additional effects include reduced sebum production (a 23%−35% decrease), diminished pore diameter (average 18%−22% reduction), and a lowered risk of rosacea. Common adverse reactions are transient erythema (lasting 3–7 days) and pain at the injection site (VAS scores of 2–4). Combining microneedling with BoNT injections enhances drug permeation efficiency and resulted in a 28% greater improvement rate for atrophic scars compared to BoNT monotherapy (45).

## 5 Surgical treatment

### 5.1 Subcision

Subcision is a technique involving the insertion of a needle beneath an acne scar to sever the underlying fibrous tissue tethering the scar. This release of the fibrous tether allows the scar to elevate. Furthermore, the induced dermal trauma stimulates neocollagenesis, which fills the depressed scar and further contributes to its elevation. A refinement of the subcision technique involves performing the procedure at two distinct tissue planes: the superficial dermis and the subcutaneous tissue (dual-plane subcision). Deeper, broader, and more pronounced rolling scars exhibit significantly greater improvement following subcision compared to smaller or shallower scars, whereas boxcar scars demonstrate substantially less improvement than rolling scars (46). Adverse effects associated with subcision include ecchymoses, bleeding, infection, and acne exacerbation.

### 5.2 Punch techniques

Punch techniques, specifically punch excision and punch grafting, are effective for elevating scar tissue in 3–4 mm acne scars, including deep boxcar scars and large ice pick scars (47). Combining punch techniques with laser therapy can help blur scar margins and enhance treatment outcomes.

### 5.3 Microneedling

Microneedling acts within the dermis to increase blood flow and stimulate the release of growth factors. This promotes the production of collagen and elastin, facilitates fibroblast migration, and reduces inflammation. Post-treatment analyses reveal increased levels of type I (48), III, and VII collagen in the skin, alongside greater thickness and randomized distribution of elastin fibers in the upper dermis. These changes collectively contribute to smoothing the skin surface in scarred areas (49).

Beyond enhancing growth factor secretion for collagen synthesis, microneedling (transcutaneous collagen induction) modulates local gene expression, thereby promoting skin repair (50). Although microneedling typically requires longer treatment intervals compared to fractional laser therapy, it demonstrates a lower incidence of post-procedural pain and hyperpigmentation—or even an absence of hyperpigmentation complications—in patients with Fitzpatrick skin types IV to VI (51).

Recent evidence indicates that combination therapies are more effective for acne scars than microneedling alone. Dual approaches combining microneedling with trichloroacetic acid (TCA), glycolic acid, or focused carbon dioxide fractional laser energy show superior outcomes. Similarly, combining non-ablative lasers with microneedling enhances efficacy (48).

Emerging studies suggest that combining oral isotretinoin therapy with early adjunctive procedures—such as chemical peels, lasers, or radiofrequency—significantly improves acne scar appearance, patient satisfaction, and quality of life (52). Furthermore, early treatment of acne scars with picosecond lasers in patients receiving low-dose oral isotretinoin is reported as a safe and effective strategy. This combined approach during early scar formation enhances scar clearance and skin barrier restoration, markedly improving patient satisfaction and quality of life without observed adverse effects in the studies (52). Consequently, concomitant dermatologic procedures are not contraindicated during oral isotretinoin therapy and do not significantly increase scar formation. While early intervention during isotretinoin therapy shows promise, large-scale RCTs are warranted to establish optimal dosing protocols and long-term safety profiles (53).

Notably, some research proposes that acne scars may not be permanent, suggesting spontaneous resolution of scars ≤ 1.5 mm within 12 weeks—indicating dynamic characteristics of atrophic scarring (54). However, as this finding stems from a retrospective, image-based analysis, potential bias exists, warranting further validation. This observation nevertheless implies that smaller acne scars (< 1.5 mm) may warrant an initial observation period before intervention.

## 6 Discussion

**Figure 2:**
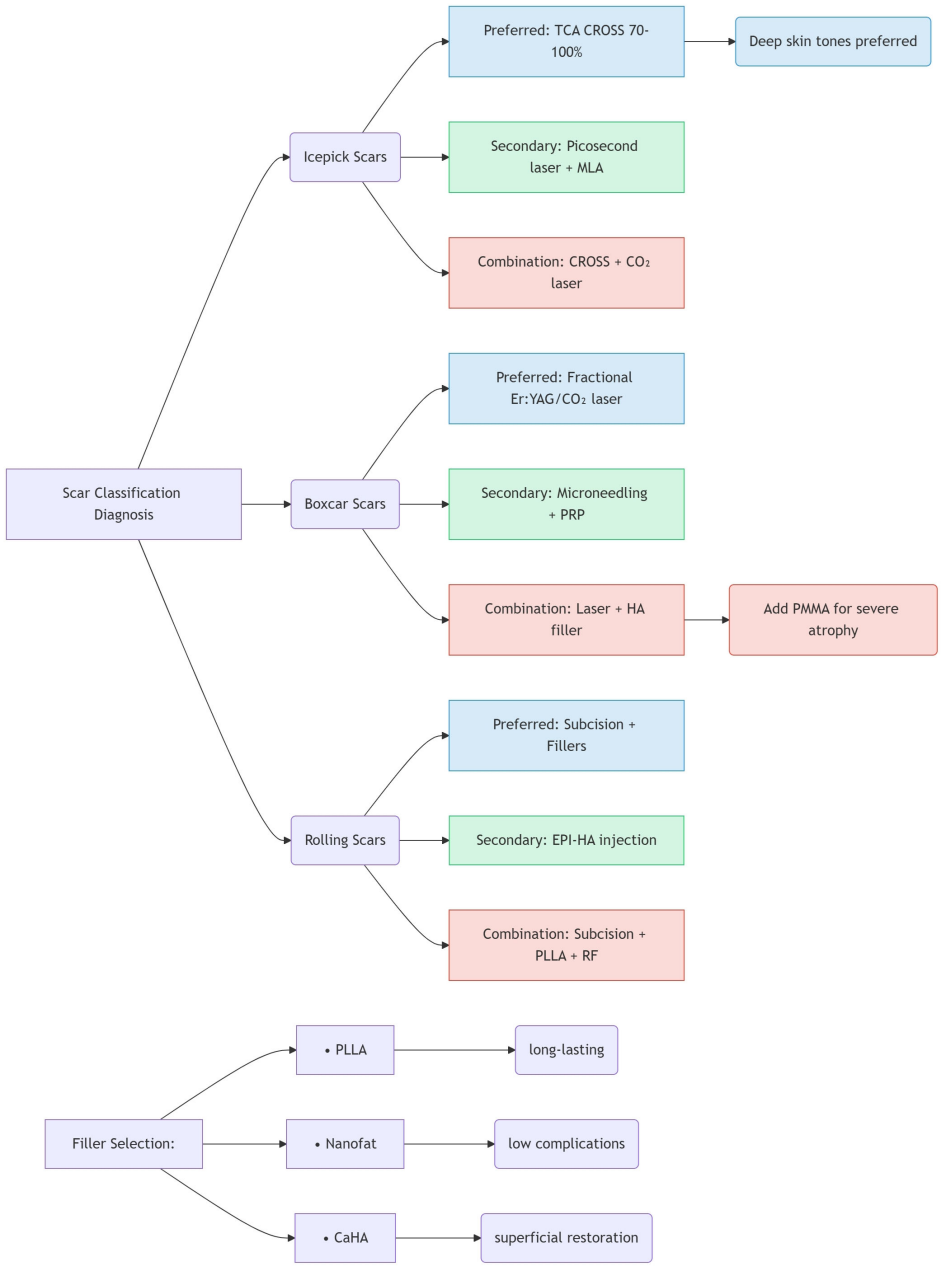
Evidence-based combination therapy algorithm.

This review synthesizes the current landscape of acne scar management, emphasizing the critical need for personalized, morphology-driven treatment strategies. The heterogeneity of scar subtypes—particularly the prevalence and distinct characteristics of atrophic scars (ice pick, boxcar, rolling)—dictates that no single modality universally suffices. Critical analysis of the current evidence reveals several key insights for acne scar management. First, an intrinsic efficacy-safety balance exists across modalities: ablative fractional lasers (CO_2_ and Er:YAG), while achieving high efficacy rates approaching 90%, carry significant risks of post-inflammatory hyperpigmentation (PIH) and prolonged erythema, particularly in darker skin types (Fitzpatrick III–VI). Conversely, non-ablative lasers and radiofrequency offer safer profiles with reduced downtime, albeit generally lower efficacy, rendering them suitable for milder scarring or sensitive patients. Picosecond lasers, especially the 1,064-nm wavelength with microlens array (MLA) technology, represent a promising middle ground, demonstrating efficacy comparable to fractional CO_2_ lasers but with a reduced PIH risk and faster recovery. Second, the limitations inherent to monotherapy underscore the clear superiority of multimodal approaches; Figure 2 provides an evidence-based clinical decision algorithm guiding morphology-driven combination strategies. Studies document synergistic effects for several principal combinations: chemical reconstruction of skin scars (CROSS) combined with energy-based devices or microneedling enhances collagen remodeling in deep scars (e.g., ice pick) while mitigating complications associated with high-concentration peels or aggressive laser monotherapy; Subcision paired with fillers (e.g., HA, CaHA, PLLA, nanofat) is essential for releasing fibrotic tethers in rolling scars prior to volume restoration—with electro-pneumatic HA injection (EPI-HA) showing particular promise for severe cases via its unique “nanobullet cutting effect”; Fractional ablative lasers combined with platelet-rich plasma (PRP) or platelet-rich fibrin (PRF) significantly improve scar revision outcomes and accelerate healing by leveraging growth factors to amplify collagen synthesis and remodeling, thereby enhancing patient quality of life; Microneedling augmented with adjuvants like trichloroacetic acid (TCA), glycolic acid, PRP, PRF, or non-ablative lasers demonstrably boosts neocollagenesis and absorption efficacy beyond microneedling alone. Third, the evolution of minimally invasive options continues, with novel filling agents (PLLA offering longevity up to 24 months, CaHA providing sustained superficial correction) and biologics (PRF enabling sustained growth factor release, autologous fibroblasts/nanofat/stem cells facilitating natural tissue regeneration) enabling effective volume restoration with favorable safety. Techniques such as EPI-HA and fractional radiofrequency further minimize invasiveness and downtime. Fourth, the importance of early intervention is increasingly recognized: emerging evidence challenges the traditional dogma of delaying procedures during oral isotretinoin therapy, demonstrating that early scar intervention using picosecond lasers, chemical peels, or radiofrequency alongside low-dose isotretinoin is safe and effective—improving scar clearance, skin barrier restoration, and patient satisfaction without increasing adverse events. While spontaneous regression of very small scars (≤1.5 mm) suggests a potential observation window, proactive early treatment for established scars yields superior functional and aesthetic outcomes. Finally, cost and accessibility considerations remain paramount despite technological advances; socioeconomic constraints significantly impact practical implementation. Although advanced modalities (e.g., picosecond lasers, autologous cell therapies) offer high efficacy, their cost can be prohibitive. Cost-effective techniques like TCA CROSS retain significant value, particularly in resource-limited settings or for specific indications such as ice pick scars in dark skin, despite recognized efficacy limitations compared to lasers. Optimizing synergistic combination strategies can enhance overall cost-effectiveness by reducing the total number of treatment sessions required compared to sequential monotherapies.

### 6.1 Limitations and future directions

While this review synthesizes current evidence for acne scar management, its primary limitation lies in the necessary conciseness of strategy descriptions. Given the vast heterogeneity of scar types (ice pick, boxcar, rolling), skin phototypes (I–VI), and treatment modalities (energy-based devices, fillers, biologics), a systematic review format inherently restricts deep technical exploration of each approach. For instance: Laser parameter optimization (fluence, density, pulse duration) could not be detailed for every scar subtype. Filler rheology (G', viscosity, extrusion force) was summarized rather than exhaustively analyzed. Molecular mechanisms (e.g., TGF-β pathway modulation by combined therapies) were streamlined for clinical applicability.

The current evidence base for acne scar management relies predominantly on cohort studies and split-face trials. To establish definitive therapeutic hierarchies, larger-scale, long-term randomized controlled trials (RCTs) directly comparing diverse combination therapies are imperative. Further research is warranted across several critical domains: to optimize radiofrequency (RF) treatment protocols and definitively establish its comparative efficacy against established laser modalities; to validate the long-term clinical outcomes and cost-benefit profiles of novel biologic agents, including platelet-rich fibrin (PRF), stem cells, and autologous fibroblasts; to confirm the dynamics and clinical relevance of spontaneous regression observed in very small scars; and to refine personalized treatment algorithms that integrate key variables such as scar morphology, skin phototype, scar chronicity, and patient-specific cost tolerance thresholds.

## 7 Conclusion

Acne scarring imposes a profound psychological burden, necessitating effective, tailored therapeutic strategies. This review affirms that successful management hinges on meticulous scar classification and the strategic integration of multiple treatment modalities. In conclusion, Future management should integrate morphology-driven algorithms (e.g., CROSS+laser for ice-pick scars, subcision+fillers for rolling scars) with early combinatorial approaches. Prioritizing patient-specific factors (skin phototype, cost tolerance) and emerging biologics (PRF, stem cells) will optimize functional and aesthetic outcomes.
